# Cross-Coupled Control for All-Terrain Rovers

**DOI:** 10.3390/s130100785

**Published:** 2013-01-08

**Authors:** Giulio Reina

**Affiliations:** Department of Engineering for Innovation, University of Salento, via Arnesano, 73100 Lecce, Italy; E-Mail: giulio.reina@unisalento.it; Tel.: +39-0832-297-814

**Keywords:** all-terrain rovers, motion control, slippage reduction

## Abstract

Mobile robots are increasingly being used in challenging outdoor environments for applications that include construction, mining, agriculture, military and planetary exploration. In order to accomplish the planned task, it is critical that the motion control system ensure accuracy and robustness. The achievement of high performance on rough terrain is tightly connected with the minimization of vehicle-terrain dynamics effects such as slipping and skidding. This paper presents a cross-coupled controller for a 4-wheel-drive/4-wheel-steer robot, which optimizes the wheel motors' control algorithm to reduce synchronization errors that would otherwise result in wheel slip with conventional controllers. Experimental results, obtained with an all-terrain rover operating on agricultural terrain, are presented to validate the system. It is shown that the proposed approach is effective in reducing slippage and vehicle posture errors.

## 1. Introduction

For mobile robots driving across challenging terrains, the greatest enemy of motion accuracy is wheel slippage. As the vehicle travels on uneven terrain, its suspension system provides for compliance with terrain topography, possibly resulting in different loading profiles for its drive wheels. Since each wheel is generally controlled independently in a closed-loop manner, this may result in one wheel speeding up while another wheel slowing down to reach its speed set point, thus promoting slippage and tendency to sideslip as the robot negotiates large obstacles. The problem is even more severe in so-called over-constrained robots, *i.e.*, featuring more independent motors than degrees of motion. A notable example are the NASA's Mars rovers [1], which are equipped with six independently controlled drive motors and six independently controlled steering motors. Other examples of mobile robots with over-constrained drive systems are the six-wheeled Shrimp [2], the Nomad Arctic traverse robot developed at Carnegie Mellon University [3], wheeled snake-type robots like the ones developed by Hirose *et al.* [4], and hybrid leg/wheel systems [5]. For all of these vehicles the designers accept the reduction of motion accuracy in their over-constrained systems as a tradeoff for enhanced mobility One way to reduce the occurrence of slippage in over-constrained vehicle is the use of a Cross-Coupled Control (CCC) strategy that can be applied without the need for mechanical redesign. This approach was originally developed by Borenstein and Koren [6] for a two-wheel differential drive vehicle; it was later refined and applied to a four-wheel drive skid-steer robot to improve odometry accuracy [7].

In this paper, the CCC idea is implemented and experimentally validated for the general case of a rover outfitted with four independently steered and driven wheels. The system continuously compares the actual encoder readings from all four wheels and issues corrective commands to the motors to slow down the motors that are faster and speed up the motors that are slower than the others. In conventional mobile robots controllers, each drive loop receives no information about the others, and any disturbance in one loop causes an error that is corrected only by this loop, while the other loops carry on as before. Cross-coupled control is used to remedy this problem by sharing the feedback information of all control loops. The overall effect is that the wheel velocities are matched more tightly even in the presence of internal and external disturbances. It should be also noted that the proposed control strategy does not require any additional sensors other than wheel encoders and steer potentiometers that are commonly available in most robots.

A cross-coupled LQR-based controller was proposed and demonstrated via simulations for a four-wheel robot in [8], aiming to drive and steer all the wheels at their respective required angles keeping the corresponding errors mutually proportional. Behavior-based approaches were also used to solve the actuators' coordination problem in four-wheel-steering robots [9,10]. Specifically in [10], the authors proposed a steering controller where virtual linkages are created between each wheel to maintain the correct kinematic constraint and minimize wheel slip. An alternative solution for the wheel synchronization problem was proposed in [11], who used a voting scheme to synchronize the six wheels of JPLs FIDO Mars Rover.

**Figure 1.**
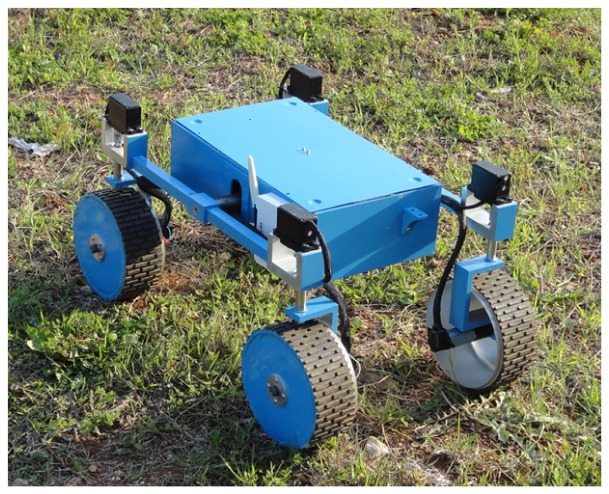
The rover test platform employed in this research.

The proposed CCC algorithm is validated in the field using the all-terrain rover Dune, built at the Applied Mechanics Laboratory of the University of Salento. Dune, shown in Figure 1, is an independently controlled four-wheel-drive/four-wheel steer mobile robot, also featuring a rocker-type suspension system. This architecture provides a high degree of mobility, allowing the robot to safely traverse rocks over one and half its wheel diameter and to perform special maneuvers such as crab and turn-on the spot motion. Its operational speed ranges from 2 to 40 cm/s. The sensor suite is composed of optical encoders and potentiometers, to measure, respectively, the wheel angular velocities and the wheel steering angles. A fiber optic gyro is also used to provide the actual rate-of-turn of the robot during experiments.

One other important benefit of the CCC system should be mentioned. As the rover travels over highly irregular terrain, it is possible the case that one wheel is “in the air”. If each of the wheels is independently powered by a motor, then the free-spinning wheel and motor do not contribute torque to propel the vehicle forward. As a result, the vehicle may stall on high-resistance surfaces, such as soft sand. The CCC partially mitigates this problem by increasing the speed of the “stuck” wheel, as the system attempts to equalize the speed of the remaining cross-coupled wheels.

The remainder of the paper is organized as follows. Basic principles of vehicle kinematics are recalled in Section 2. Section 3 describes the proposed motion controller in detail. In Section 4, the system is validated in the field with the rover Dune and its performance are compared against the conventional approach. Section 5 concludes this paper.

## 2. Kinematic Modeling

**Figure 2.**
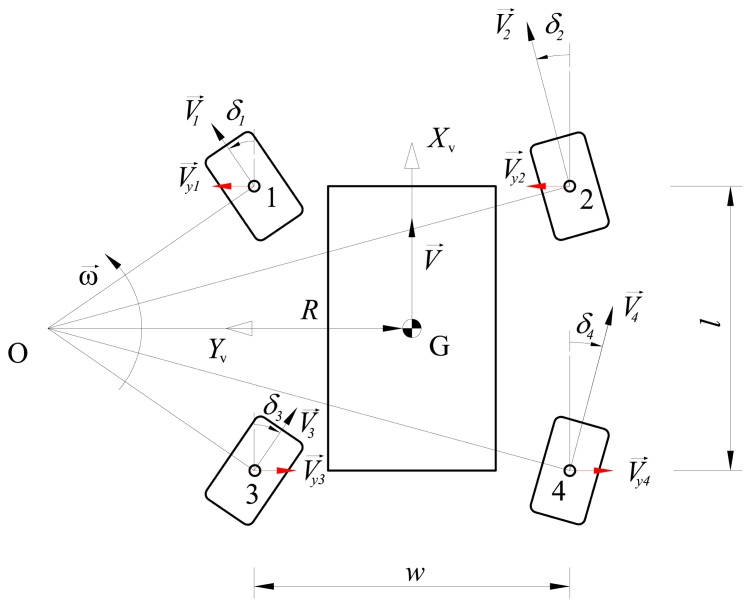
A four-wheel-drive/four-wheel steer mobile robot. Note that all velocity vectors are drawn to scale.

Consider a four-wheel-drive/steer robot that is turning counter-clockwise, as shown in Figure 2, under the assumption of planar motion. Typical travel speeds for all-terrain rovers are low and the kinematic condition that the perpendicular lines to each wheel meet at one point must be applied in order to guarantee slip-free turning. The intersection point *O* is the turning center or instantaneous center of rotation of the vehicle. It may change from moment to moment; for straight-line motion, the radius from *O* to each wheel is of infinite length, whereas it is null for turn-on-the spot motion. The rover mass center *G* turns on a circular path with radius *R*, and linear and angular velocity vector *V⃗* and *ω⃗*, respectively. The distance between the front and the rear axle is the wheelbase *l*, whereas the distance between the wheels of the same axle is called the track *w*. Each wheel has a linear velocity vector *V⃗_i_* and a steering angle *δ_i_*, which is measured between the longitudinal direction of the vehicle and the steering direction of the wheel. The vector projection of the speed vector *V⃗_i_* onto the *y*-axis of the vehicle is called lateral velocity component *V⃗_y,i_* and it is marked in red in Figure 2. The concept of lateral velocity component will be useful later in the paper to define the cross-coupled control strategy during turning maneuvers. In this work, a symmetric four-wheel steering rover is considered where the front and rear wheels steer opposite to each other equally. According to the notation used in this paper, vector quantities are distinguished from scalar ones by using a right-pointing arrow above their names.

## 3. The Cross-Coupled Control

Mobile robots can be considered as multi-axis drive servomechanisms. Disturbances that affect one control loop may differ from disturbances that affect the other loops. Even if each axis is equipped with a high performance tracking controller, the error in one axis will affect the whole motion of the system. If this phenomenon is overlooked and each axis is controlled independently, it will most likely lead to poor motion accuracy. The problem is exacerbated in over-constrained vehicles, where any momentary mismatch between wheel velocities with respect to the vehicle kinematic model will result in wheels “fighting” each other with consequent ill-effects, including increase in power consumption and errors in the odometry-based position estimation [12,13], and reduction in traction and climbing ability [14]. For instance, during a simple forward-backward motion all the wheels have to run at exact same speed to avoid slippage, any momentary mismatch between wheel velocities will force the wheels to skid or slip unpredictably.

In order to achieve a coordination control, a cross-coupled control approach can be applied where the whole multi-axis system is considered as a single system. Compensations are calculated by taking into account the mutual influences to match more tightly the different axes and consequently reduce the error in the global motion control. The CCC continuously compares the wheel encoder pulses of a robot to slow down the motor that is faster and speed up the motor that is slower than the others. The overall effect of this approach is that the wheel velocities are matched more tightly according to the correct kinematic behavior of the robot. The proposed cross-coupled approach differentiates between straight and turning motion. When the rover follows a straight path, the average longitudinal velocity is estimated and the deviation of a given wheel from this average value is used to adjust the associated set point in order to gain speed synchronization. During turning maneuvers, the single speed set points are corrected based on the discrepancy with a so-defined mean absolute lateral velocity. Details of how the CCC handles these two types of motion are presented in the remainder of this section.

### 3.1. Straight Motion

**Figure 3.**
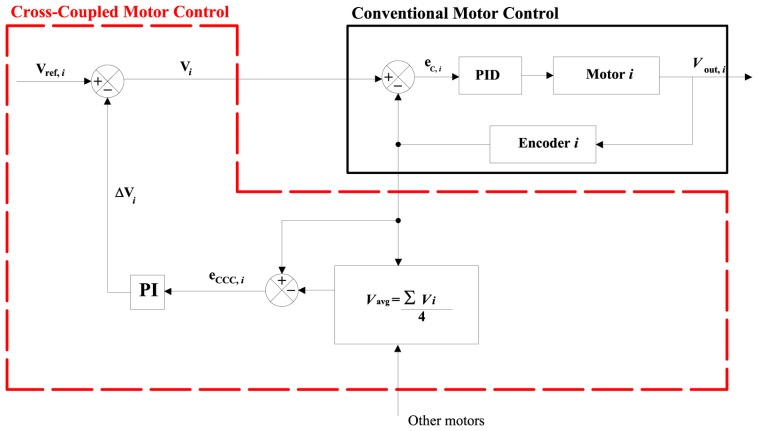
Block diagram of a cross-coupled control loop for the *i*-th wheel of the rover during straight motion. The conventional part of the control loop is denoted by a solid black line. The CCC part is outlined by a red dashed line.

Figure 3 shows a block diagram with the implementation of the cross-coupled control for one wheel of the rover. The scheme is the same for the remaining wheels and it is not reported. Based on the estimation of the wheel velocities *V_i_* (*i* = 1,…, 4) by encoders, the vehicle speed can be calculated by

(1) $$V_{\text{avg}}=\frac{\overset{4}{\underset{i=1}{\text{∑}}}V_{i}}{4}$$

The deviation of the *i*-th wheel from the average value, *e_CCC_*,*_i_* = *V_i_* − *V_avg_*, is used as input error to a proportional-integral (PI) controller that estimates the required change in the commanded velocity set point Δ*V_i_* relative to the nominal velocity set point *V_ref,i_* according to the following equation:

(2) $$V_{i}=V_{\text{ref},i}-\Delta V_{i}$$

### 3.2. Turning Motion

**Figure 4.**
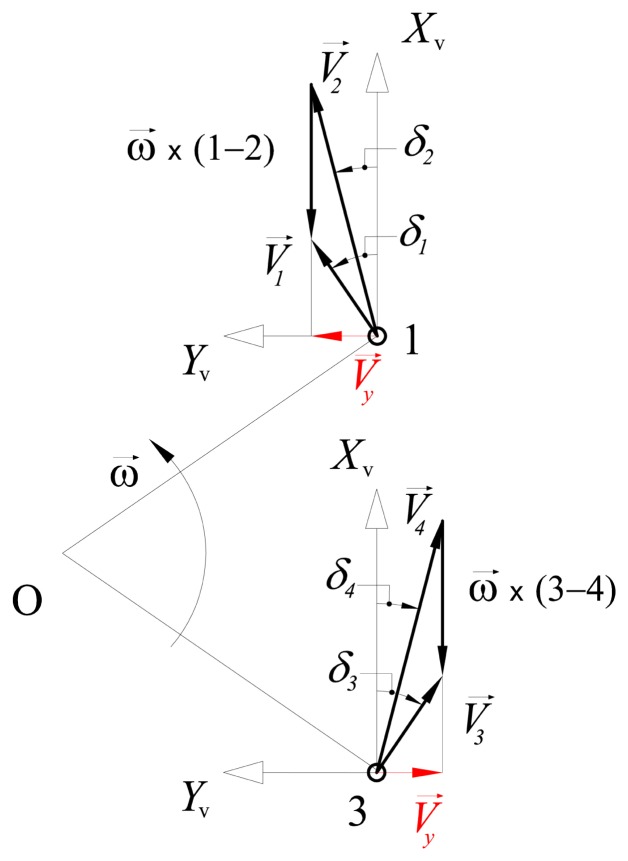
Graphical analysis of the wheel velocities of a rover with symmetric steering. All wheels admit equal lateral velocity components in magnitude. Direction of the lateral velocity components of the front wheel pair is opposite to that of the rear pair.

During turning maneuvers, each wheel travels with a velocity proportional to the distance from the instantaneous center of rotation, *i.e.*, the inner wheels are slower than the outer wheels, as shown in Figure 2. However, the projection of wheel velocities along the *y*-axis of the vehicle must be equal in absolute value in order to ensure a correct kinematic behavior. This can be proved via graphical velocity analysis of the rover, as illustrated in Figure 4. The vector relationships between the velocities of the front and rear wheel pairs are respectively

(3) $${\vec{V}}_{1}={\vec{V}}_{2}+\vec{\omega }\times (1-2)$$

(4) $${\vec{V}}_{3}={\vec{V}}_{4}+\vec{\omega }\times (3-4)$$

where *ω⃗* is the rate of turn of the rover and the geometric point *I* = 1,…, 4 refers to the center of the associated wheel. If Equations (3) and (4) are projected over the *y*-axis of the rover, it gets

(5) $$V_{y,f}=V_{1}\cdot \sin\delta_{1}=V_{2}\cdot \sin\delta_{2}$$

(6) $$V_{y,r}=V_{3}\cdot \sin\delta_{3}=V_{4}\cdot \sin\delta_{4}$$

Under the assumption of symmetric steering, the magnitude of the lateral velocity component has to be equal for the front and rear wheel pairs, *i.e.*, *V_y,f_* = *V_y,r_* = *V_y_*. Note, however, that the direction of the lateral velocity component for the front wheels is opposite to that of the rear wheels.

**Figure 5.**
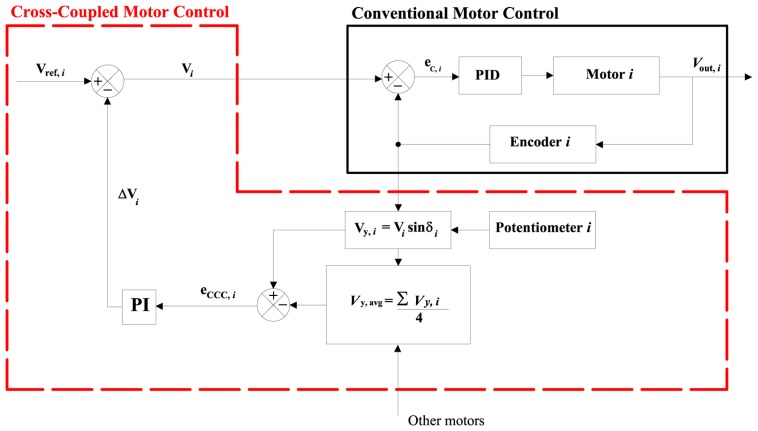
Block diagram of a cross-coupled control loop for a generic turning maneuver. The conventional part of the control loop is denoted by a solid black line. The CCC part is outlined by a red dashed line.

Figure 5 shows the typical block diagram with the implementation of the cross-coupled control for one wheel of the rover.

The lateral velocity component for wheel *i* can be obtained based on the estimation of its linear velocity *V_i_* by encoder, and steer angle *δ_i_* by potentiometer

(7) $$V_{y,i}=V_{i}\cdot \sin\left\|\delta_{i}\right\|$$

Since we are interested in the magnitude of *V_y,i_*, the absolute value of *δ_i_* is considered. Then, the mean absolute lateral velocity can be obtained by

(8) $$V_{y,\text{avg}}=\frac{\overset{4}{\underset{i=1}{\text{∑}}}V_{y,i}}{4}$$

And the deviation of the *i*-th wheel from *V_y,avg_*, *e_CCC_*,*_i_* = *V_y,i_* − *V_y,avg_*, is used as input error to the CCC correction module following the same rationale described for straight-line motion. The new velocity set point is obtained again using Equation (2). This approach can be applied to any turning maneuver including turning on the spot. It should be also noted that the CCC does not provide any correction for the steering angles. This choice is motivated by the observation that the traction dynamics is significantly faster than the steering one. Therefore, traction and steering control are tackled separately with decoupled control loops.

## 4. Experimental Results

**Figure 6.**
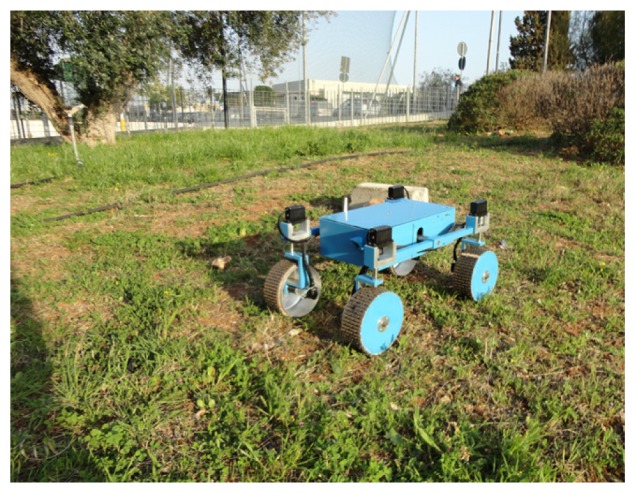
The test field and the rover Dune.

In this section, experimental results are presented to validate the proposed cross-coupled control approach. The system was integrated with the rover Dune (see Figure 1) and demonstrated in a rural environment at a University of Salento's test facility. The test field, shown in Figure 6, was mainly composed of relatively even agricultural terrain with sparse low grass and rocks of small-medium size. During the experiments, the rover was remotely driven by a human operator as the onboard computer gathered encoder, potentiometer and gyroscope information for subsequent off-line analysis.

**Figure 7.**
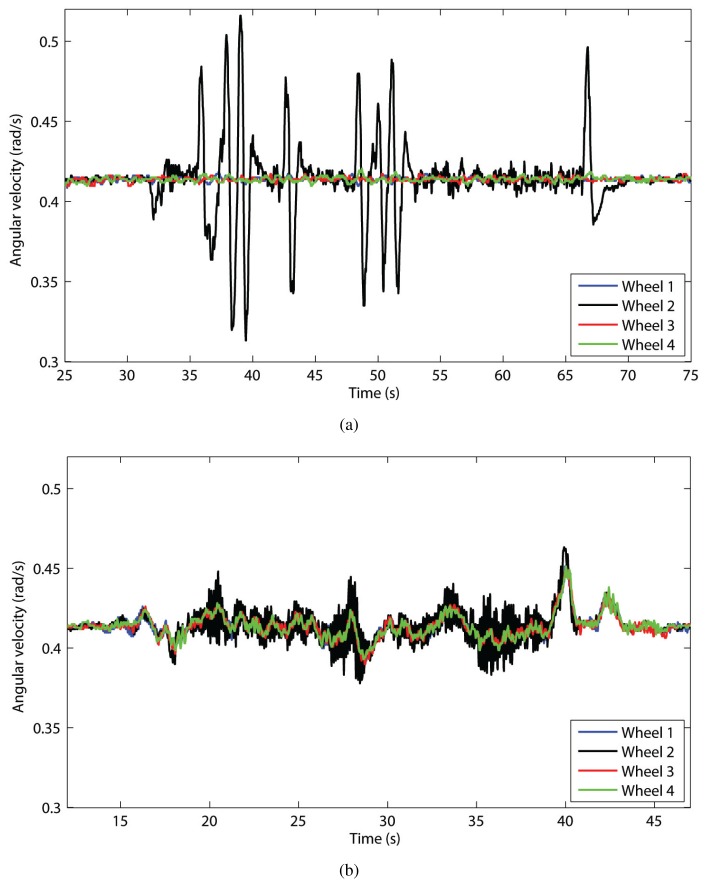
Change in wheel angular velocities in presence of external disturbances applied to Wheel 2. (**a**) Conventional control: The control loops do not share information resulting in Wheel 2 rotating faster or slower than the other wheels. (**b**) Cross-coupled control: By sharing information between the control loops, all wheel velocities are matched more tightly.

The main idea behind the cross-coupled approach can be explained with a preliminary experiment where the rover was commanded to drive straight forward as braking torques were applied to one of the wheels, *i.e.*, Wheel 2, in order to introduce random disturbances into its control loop. As shown in Figure 7(a), under conventional controller (CC) large differences in the wheel velocities were produced with consequent, undesired increase in the amount of slippage. This effect is due to the lack of communication between the control loops. The conventional control was implemented as a standard Proportional-Integral-Differential (PID) controller, as customary in many robotic platforms. The application of the cross-coupled control strategy improved the rover's behavior as shown in Figure 7(b). In contrast to conventional control, the cross-coupled controller compares all control loops instant by instant (a sampling interval of *T* = 0.01 s was used) decreasing the wheel velocities that are higher and increasing the wheel velocities that are lower. The overall result is a better synchronization of all wheel velocities with a consequent minimization of slippage.

Different types of experiments were conducted on Dune to evaluate the performance of the CCC method with respect to the conventional control. Two main performance metrics were used to quantitatively evaluate the system: the orientation error and the tracking error. The orientation error is defined as

(9) $$\Delta \theta =\frac{\Delta d_{r}-\Delta d_{l}}{w}$$

where Δ*d_r_* and Δ*d_l_* are the right and left side longitudinal displacement as measured by encoders in the sampling period and *w* the track of the vehicle. Feng *et al.*[15], consider Δ*θ* as the most significant error, *i.e.*, the error that has the largest impact on motion accuracy. It has been shown to play a fundamental role in dead-reckoning accuracy since it grows with the traveled distance.

The tracking error can be defined for wheel *i* as

(10) $$\Delta t_{i}=\left(\frac{V_{i}}{V_{\text{avg}}}-1\right)\times 100\%$$

For example, if one wheel spins at high angular velocity without the equivalent translatory progression of the rover, *V_i_* ≥ *V_avg_*, a positive value for the tracking error results. Δ*t_i_* can be interpreted as a measure of the effectiveness of the control system in avoiding slippage.

Two types of experiments were considered.

1. Paths generated by single straight-line and turning maneuvers.
2. Closed loop paths, where both straight lines and 90° turns in the four corners of the rectangular path contributed to the overall errors.

### 4.1. Single Primitives of Motion

**Figure 8.**
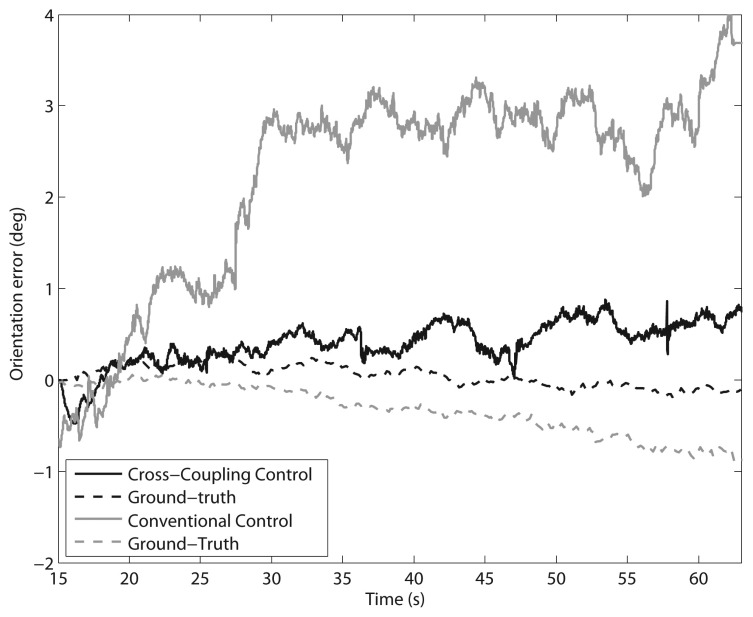
Comparison of cross-coupled control with conventional control: Change in the orientation error during a single 5-meter straight path test on agricultural terrain (V = 10 cm/s). Black solid line: Encoder-derived orientation error using the CCC. Black dashed line: Gyro-derived ground truth. Grey solid line: Encoder-derived orientation error using the CC. Grey dashed line: Gyro-derived ground truth.

Tests were first performed along a 5-meter straight path with a constant travel speed of 10 cm/s. Six runs with each control method were performed. Figure 8 shows a comparison of the typical orientation error growth using the CC (marked by a solid grey line) and the CCC (denoted by a solid black line) controllers in a sample run. The encoder-derived measurement (solid line) is compared with the actual heading change (dashed line), provided by a fiber-optic gyro aligned along the vertical axis of the vehicle that is used as the ground truth in this type of experiments. In order to gain an indication of the error accumulation, the final orientation error is considered as obtained from the odometry at the end of each run. The mean final orientation error and standard deviation in all experiments resulted in 3.8 ± 0.8 deg under CC, and 0.7 ± 0.2 deg using CCC. It is apparent from these results that using a conventional PID controller the orientation error continuously increases, while, under cross-coupled control, a correction signal is generated when discrepancies between the wheel velocities are detected, thus reducing the rover orientation error.

In order to evaluate the effectiveness in reducing slippage using the CCC, the mean tracking error was estimated for each run. Then, the average over the six experiments was calculated and collected in Table 1 along with its statistical spread. Using the CC, the average wheel slip was 0.93%, with Wheel 1 experiencing the highest value of 1.17%. The application of the CCC greatly reduced the percentage of wheel slip to only 0.23% on average with an upper bound of 0.29% for Wheel 1.

**Figure 9.**
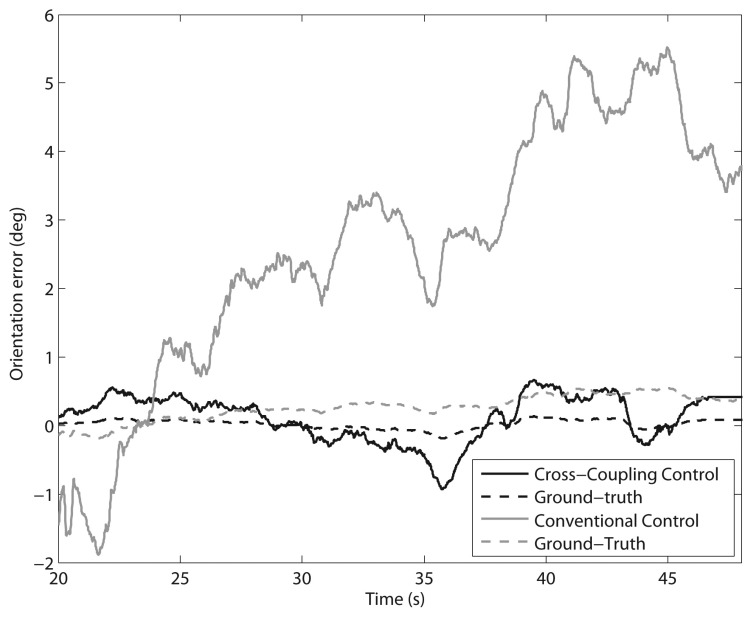
Comparison of cross-coupled control with conventional control: Change in the orientation error during a single 5-meter straight path test on asphalt road (V = 10 cm/s). Black solid line: Encoder-derived orientation error using the CCC. Black dashed line: Gyro-derived ground truth. Grey solid line: encoder-derived orientation error using the CC. Grey dashed line: Gyro-derived ground truth.

The advantage of the CCC system is also confirmed when considering higher traction surfaces. Figure 9 shows the results of a typical 5-meter straight path test performed on road asphalt with a constant travel speed of 10 cm/s, using the CC (marked by a solid grey line) and the CCC (denoted by a solid black line) controllers. Again, ground truth was provided by a fiber-optic gyro (dashed lines). Using the conventional approach, the orientation error growth is much higher than when adopting cross-coupled control that, in contrast, ensures tight synchronization of all wheel velocities.

**Figure 10.**
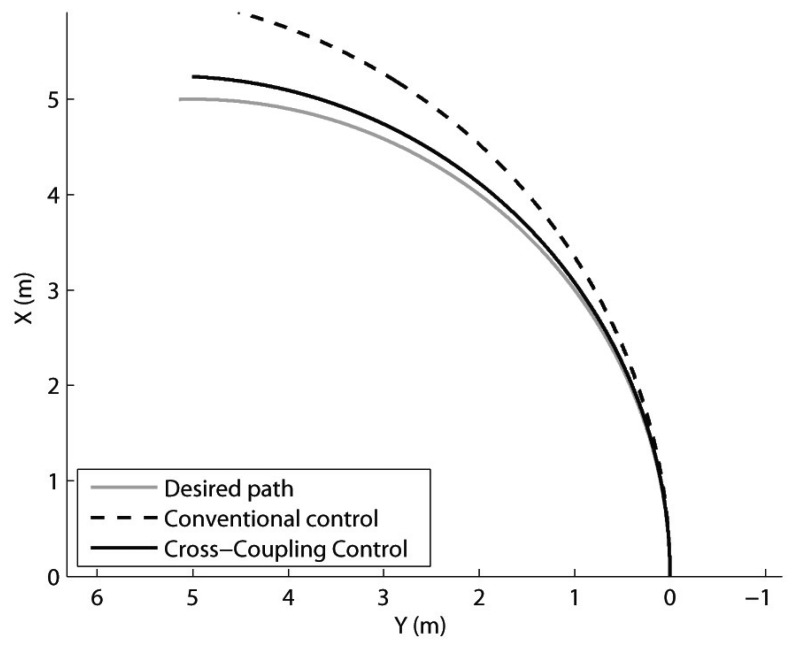
Comparison of Cross-coupled Control with Conventional Control during turning motion on agricultural terrain (V = 5 cm/s, *ω* = 0.05 rad/s). Grey solid line: Desired path. Black solid line: Encoder-derived path using the CCC. Black dashed line: Encoder-derived path using the CC.

A second set of experiments was performed driving the rover with a constant linear velocity of 5 cm/s along a circular path with a turning radius of 5-meter (*i.e.*, rate-of-turn *ω* = 0.05 rad/s) on agricultural terrain. Figure 10 shows the odometry-derived path for a sample run. The commanded path is denoted by a grey solid line, the path derived by the odometry system using the CCC is marked with a black solid line, and the CC is denoted by a dashed black line. Six similar runs with each control method were performed. The final orientation error with respect to the actual heading change of 90 deg was estimated for each run. It resulted in an average of 12.10 ± 2.10 deg using CC, and in 1.10 ± 0.21 deg on average when the CCC was applied. Wheel slip obtained as an average value over the six runs is also collected in Table 2. Under CC, the average tracking error resulted in 2.21%. The application of the CCC improved the rover performance by reducing the percentage of wheel slip to only 0.55% on average. The cross-coupled approach again outperformed the conventional control.

It is apparent from these results that the CCC module is indeed effective in minimizing the difference in wheel velocities of the rover in both straight and turning maneuvers.

### 4.2. Closed-Path Experiments

**Figure 11.**
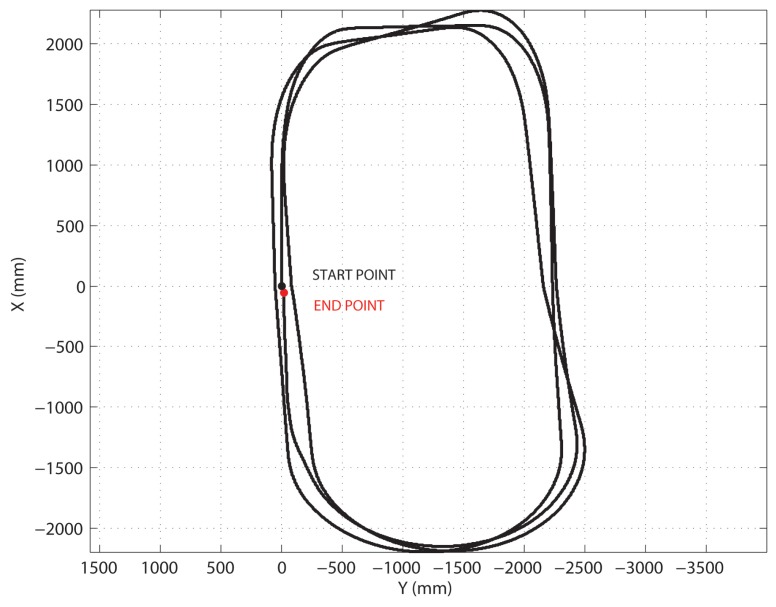
Path of the rover in a typical closed-loop run as derived by gyro-odometry, *i.e.*, linear and angular displacements were obtained by encoders and gyro, respectively

Experiments were performed along a closed path to test the improvement in motion accuracy using the CCC method. The experimental benchmark proposed in Ojeda *et al.* [16,17], was adopted. Dune traveled autonomously along a preprogrammed, near rectangular path over agricultural terrain. In order to perform long duration tests, each run consisted of three uninterrupted loops, resulting in a total travel distance of about 37 m, and a total of 1,080 degrees of turning per run, as shown for a typical test in Figure 11. The speed in all runs was 10 cm/s, and the rover did not stop before turning. A set of multiple runs was performed consisting of six runs in clockwise (CW) and six runs in counterclockwise (CCW) direction. For each test, Dune started at a marked location and ended back at the initial starting position. The discrepancy between the actual stopping position and the starting position, measured with a tape measure, is the so-called “return position error”. The average absolute positioning errors *E_x_* and *E_y_* can be obtained as

(11) $$E_{x}=\frac{1}{m}\sum_{i=1}^{m}\left\|x_{r}\right\|$$

(12) $$E_{y}=\frac{1}{m}\sum_{i=1}^{m}\left\|y_{r}\right\|$$

where *x_r_* and *y_r_* are the return position error measured along *x*- and *y*-direction, respectively, and *m* = 6 the number of runs. Finally, an absolute error can be estimated as

(13) $$E=\sqrt{E_{x}^{2}+E_{y}^{2}}$$

**Figure 12.**
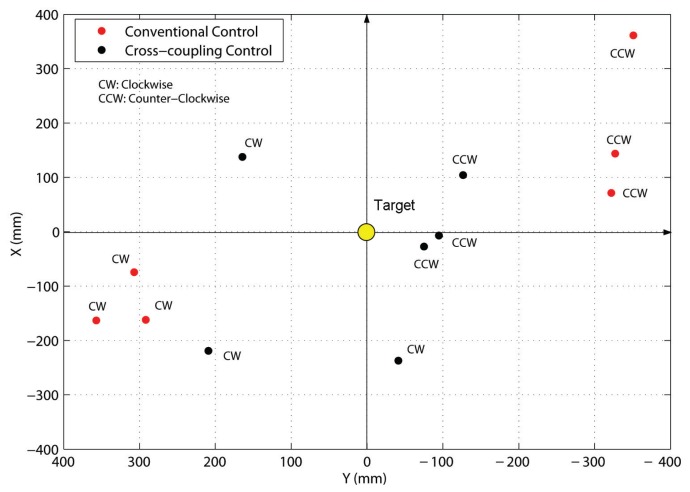
Return position errors for the closed-loop experiments. Black dot: Cross-coupled control. Red dot: Conventional control.

Figure 12 shows the return position error for each run in graphical form. Using the CCC approach, the average absolute positioning errors resulted in *E_x_* = 17.25 cm and *E_y_* = 19.41 cm, respectively, with an absolute error *E_CCC_* = (17.25, 19.41) = 25.96 cm corresponding to the 0.70% of the total path length of 37 meter associated with each run. Under conventional control the errors grew up to *E_x_* = 20.43 cm and *E_y_* = 35.23 cm with a global error *E_CC_* =(20.43, 35.23) = 40.72 cm corresponding to the 1.10% of the path length. The experimental results show that the CCC approach leads to a substantial improvement in motion accuracy than conventional methods.

## 5. Conclusions

In this paper, a cross-coupled control approach was described to improve motion accuracy of all-terrain rovers with independently steered and driven wheels. It aims to optimize the motors' control algorithm of the robot to reduce synchronization errors that would otherwise result in wheel slip with conventional controllers. Experimental results obtained using a test platform, operating outdoor in a rural environment, validated the system. It was demonstrated that during single straight-line and turning maneuvers, the orientation error and the tracking error were significantly reduced to maximum values of 1.5 deg and 0.55%, respectively, which is a remarkable result when compared with the reference of more than 12 deg of orientation error and 2.21% of wheel slip. Furthermore, in closed loop experiments, the CCC showed its effectiveness in improving motion accuracy keeping the return positioning error to well under 1% of the total travel distance. This technique can be successfully applied to enhance the degree of mobility and autonomy of all-terrain robots.

## Figures and Tables

**Table 1.** Tracking error for the rover Dune during 5-meter straight-line experiments on agricultural terrain (V = 10 cm/s).

	Tracking error Δ*t*	
	Conventional control (%)	Cross-coupled control (%)
Wheel 1	1.17 ±0.15	0.29 ± 0.03
Wheel 2	0.75 ±0.10	0.19 ±0.02
Wheel 3	0.94 ±0.12	0.23 ± 0.03
Wheel 4	0.86 ±0.10	0.21 ± 0.02

**Table 2.** Tracking error for the rover Dune during a turning maneuver (*V* = 5 cm/s, *ω* = 0.05 rad/s).

	Tracking error Δ*t*	
	Conventional control (%)	Cross-coupled control (%)
Wheel 1	2.46 ± 0.44	0.62 ±0.10
Wheel 2	2.48 ± 0.41	0.62 ± 0.08
Wheel 3	1.97 ± 0.37	0.49 ± 0.06
Wheel 4	1.95 ± 0.38	0.48 ± 0.06
